# Overview and Update on Methods for Cargo Loading into Extracellular Vesicles

**DOI:** 10.3390/pr9020356

**Published:** 2021-02-15

**Authors:** Yohan Han, Timothy W. Jones, Saugata Dutta, Yin Zhu, Xiaoyun Wang, S. Priya Narayanan, Susan C. Fagan, Duo Zhang

**Affiliations:** 1 Clinical and Experimental Therapeutics, College of Pharmacy, University of Georgia and Charlie Norwood VA Medical Center, Augusta, GA 30912, USA;; 2 Center for Vaccines and Immunology, University of Georgia, Athens, GA 30602, USA;; 3 Vascular Biology Center, Augusta University, Augusta, GA 30912, USA; 4 James and Jean Culver Vision Discovery Institute, Augusta University, Augusta, GA 30912, USA

**Keywords:** drug delivery, exosome, microvesicle, apoptotic body, small RNAs

## Abstract

The enormous library of pharmaceutical compounds presents endless research avenues. However, several factors limit the therapeutic potential of these drugs, such as drug resistance, stability, off-target toxicity, and inadequate delivery to the site of action. Extracellular vesicles (EVs) are lipid bilayer-delimited particles and are naturally released from cells. Growing evidence shows that EVs have great potential to serve as effective drug carriers. Since EVs can not only transfer biological information, but also effectively deliver hydrophobic drugs into cells, the application of EVs as a novel drug delivery system has attracted considerable scientific interest. Recently, EVs loaded with siRNA, miRNA, mRNA, CRISPR/Cas9, proteins, or therapeutic drugs show improved delivery efficiency and drug effect. In this review, we summarize the methods used for the cargo loading into EVs, including siRNA, miRNA, mRNA, CRISPR/Cas9, proteins, and therapeutic drugs. Furthermore, we also include the recent advance in engineered EVs for drug delivery. Finally, both advantages and challenges of EVs as a new drug delivery system are discussed. Here, we encourage researchers to further develop convenient and reliable loading methods for the potential clinical applications of EVs as drug carriers in the future.

## 1. Introduction

Multicellular organisms communicate intercellularly via soluble cell-secreted factors, and the direct interactions between cell surfaces are an essential part of exchanging genetic information. Distant cell–cell communication in eukaryotic cells utilizes membrane-derived vesicles secreted into the extracellular space [1]. Extracellular vesicles (EVs), nano-sized secreted cellular particles, were first purified and validated from various cells during their discovery in the 1980s. EVs were considered simple membrane fragments until Raposo et al. characterized the antigen-presenting EVs secreted by B lymphocytes in 1996 [2]. Generally, EVs are classified into exosomes, microvesicles, and apoptotic bodies according to their diameters and intracellular origins [3,4]. Exosomes are the smallest EVs with a size of 40–100 nm. They originate from multivesicular endosomes (MVEs), which contain intraluminal vesicles (ILVs) incorporated with RNA, DNA, proteins, and lipid. MVEs exit the cell through plasma membrane fusion, which allows intracellular molecules to traffic through extracellular spaces to distant cells. While microvesicles with a size of 50–1000 nm are derived directly from the plasma membrane, apoptotic bodies are typically 1000–5000 nm in diameter and secreted from the cells under apoptosis [3,4]. Although each of them has specific characteristics and forming processes, distinguishing them experimentally presents a challenge due to their overlapping sizes and lack of distinct subtype markers.

The effects of EVs and their cargo have been studied in many diseases such as cancer [5], cardiovascular diseases [6], autoimmune diseases [7], inflammatory diseases [8], and type 2 diabetes mellitus [9]. Their usability and effectiveness are also under evaluation in clinical trials [10]. Therapeutic agents, including siRNA [11], miRNA [12], mRNA/proteins [13], CRISPR/Cas9 [14], and chemical drugs [15] were loaded into EVs for treating diseases. It has been reported that an exosome-based delivery system could target cancer stem cells with improved efficiency and specificity [16]. Although technical difficulties still exist in EVs preparation and cargo-loading efficiency, the therapeutic platform of EVs has promise as a novel drug delivery method. Considering their potential applications for human diseases, we summarize not only the EVs loading methods, but also introduce the advantages and challenges of these loading methods.

## 2. EV Loading Methods

Cargos can be packaged into EVs with or without the help of donor cells. Thus, methods for encapsulating cargo into EVs can be roughly divided into two types: cell-based loading methods and non-cell-based loading methods. In the cell-based loading approach, cargos are usually delivered into the donor cells first. After being packaged into EVs, the cargos can be secreted and collected in an EV-carrying manner for therapeutic use [17]. The non-cell-based loading approach involves directly loading chemical or biomolecules into isolated EVs through electroporation, sonication, incubation, and/or transfection [18]. In the following content, these methods are reviewed according to the cargos.

### 2.1. Cell-Based Loading Methods

**Figure 1.**
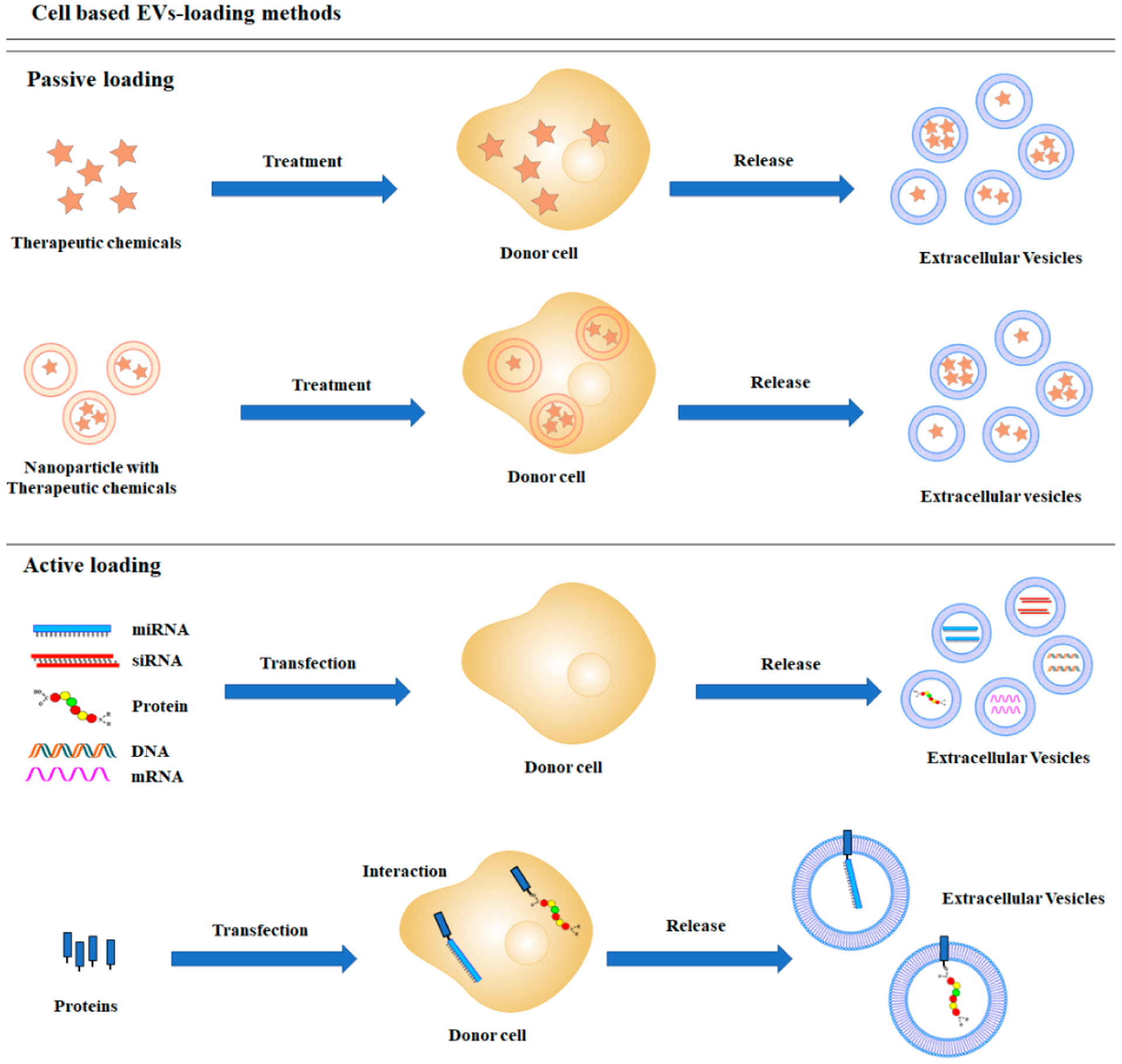
Schematic diagrams of cell-based cargo loading methods.

Cell-based loading, also known as endogenous loading, employs the indirect deposition of a therapeutic cargo into EVs through donor cell manipulation (Figure 1). Incubation and transfection are widely used to load the cargo into donor cells. After purification of released EVs, EV-carrying cargos can be used for therapeutic purposes [19–21]. This method provides a convenient and effective way for loading biological materials and drug therapies into EVs.

#### 2.1.1. Doxorubicin

The chemotherapeutic agent doxorubicin is both highly efficacious and cytotoxic, specifically to cardiac cells. Efficacy has been limited by insufficient cellular uptake, instability in the acidic tumor environment, and intermediate solubility due to amphoteric property [22,23]. EVs can mitigate these limitations, and doxorubicin can be loaded into EVs simply by application to cells in vitro. Jang et al. and Kanchanapally et al. treated various cells with doxorubicin, including U937 monocytes, macrophages, and cancer cells. After incubation for 24 to 48 h, doxorubicin-loaded EVs could be isolated from culture media [24,25]. Silva et al. developed hybrid EVs, which consist of components of macrophage-derived EVs and iron oxide nanoparticles. This team treated THP-1 macrophages with magnetic iron oxide nanoparticles and doxorubicin, which results in the generation of EVs containing both iron oxide and chemotherapeutic agents. These hybrid EVs could be manipulated by magnetic force for delivering the doxorubicin to the cancer cells [23]. Moreover, Yong et al. used an electrochemical etching method to load doxorubicin into luminescent porous silicon nanoparticles (PSiNPs) for cell treatment. Through this process, exosome-sheathed PSiNPs (E-PSiNPs) containing doxorubicin were constructed as the drug delivery system [26].

#### 2.1.2. Curcumin

Currently, it has been demonstrated that natural products have beneficial effects for multiple human diseases. However, their commercial development is limited due to low biocompatibility, poor solubility, and poor absorption [27]. Curcumin, a natural polyphenolic compound, has been shown to suppress cancer cell growth both in vitro and in vivo (e.g., tumor stem cells). However, clinical curcumin use is problematic due to its low solubility and cellular uptake [28]. Perteghella et al. sought to remove these barriers by loading curcumin into EVs. They used silk nanoparticles to enhance loading efficiency. The nanoparticles were conjugated with curcumin and then applied to mesenchymal stem cells. After incubating for several days, EVs containing silk/curcumin nanoparticles were observed [29].

#### 2.1.3. Other Drugs

Besides the doxorubicin, Silva et al. loaded tissue-plasminogen activator (t-PA), disulfonated tetraphenyl chlorin (TPCS2a), and, 5,10,15,20-tetra(m-hydroxyphenyl)chlorin (mTHPC) into magnetic iron oxide hybrid EVs by adding the magnetic nanoparticles and drugs to the differentiated THP-1 cells. They also showed that magnetically engineered-EVs did not disturb the cellular uptake of drugs and the uptake of engineered EVs by cancer cells could be kinetically modulated and spatially controlled under a magnetic field [23]. Millard et al. loaded mTHPC into EVs isolated from human umbilical vascular endothelial cells (HUVEC). To isolate EVs containing mTHPC from cell-culture media, they treated HUVECs with mTHPC for 2 h and maintained the cells in fresh media for 72 h [30].

#### 2.1.4. miRNA

miRNA is a group of small non-coding RNA containing ~22 nucleotides that bind to the 3′-UTR of target mRNA to suppress gene expression [31]. The role of altered miRNAs has been characterized in many pathologies, such as viral infections, inflammatory diseases, and cancer [32,33]. Currently, transfection is a reproducible method for miRNAs loading into EVs through a cell-based method. For example, the transient transfection of miRNAs into donor cells can package miRNAs as a cargo into secreted EVs. Through this simple transfection, EV-loaded miRNAs such as miR-1-1, miR-210, miR-584, and miR-21-5p have shown therapeutic effects by binding to disease-induced mRNA [34–37]. On the other hand, stable transfection has also been used to generate miRNA-loaded EVs. For example, Monfared et al. generated a miR-21 expressing stable cell line using HEK 293T cells. miR-21-loaded EVs were isolated from culture media and applied therapeutically in a glioblastoma rat model [38]. Zeh et al. made an immortalized human amniocyte cell line, stably releasing EV-encapsulated-miR-493 or miR-744 [39]. Furthermore, lentiviral packaging systems can overexpress miRNAs in the donor cells, which are difficult to transfect, such as stem cells, lymphocytes, and some cancer cells. After lentiviral transduction, miR-335, miR-302a, miR-3188, miR-101-3p, and miR-125-5p were successfully encapsulated into donor cell EVs [40–45].

#### 2.1.5. siRNA

siRNA is a powerful tool to block gene expression. Different from permanent genome editing technology, siRNA inhibits gene expression at the post-transcriptional level. This specificity enables tight control of siRNA-based therapies [46]. However, the delivery of negatively charged siRNA throughout the cell membrane is challenging, and prolonged siRNA stability is also a concern [47]. EVs present a solution to these therapeutic issues inherent to siRNA. Similarly, transfection is an effective way for packaging siRNA into EVs via donor cells. For example, Zhang et al. found that transfected siRNA can be encapsulated into secreted EVs. Subsequently, they were able to identify hepatocyte growth factor (HGF) siRNA loaded EVs after donor cell transfection [48].

#### 2.1.6. mRNA/Protein

Even though mRNA-based gene therapy possesses many advantages regarding easy manipulation and expression, the unstable structure and ubiquitous presence of RNase are obstacles to overcome [49]. EVs could serve as an ideal carrier for functional mRNA delivery [50]. Given the larger size of mRNA, the development of mRNA as EV cargo can be more difficult compared with miRNA and siRNA, since most full-length mRNAs in EVs are smaller than 1 kb [51]. Thus, the length of desired mRNAs must be taken into consideration for packaging into EVs. Mizrak et al. first reported the therapeutic potential of EV-encapsulated mRNA/protein. They transfected prodrug-converting enzyme cytosine deaminase (CD)-uracil phosphoribosyltransferase (UPRT) into HEK 293T cells and isolated EVs loaded with CD-UPRT-EGFP mRNA/protein [52]. Kanada et al. observed luciferase loading of EVs isolated from luciferase transfected HEK 293FT cells [17]. Vituret et al. loaded cystic fibrosis transmembrane conductance regulator (CFTR) glycoprotein and its encoding mRNA into cystic fibrosis cells to improve the function of CFTR chloride channel. They isolated EVs from CFTR-positive Calu-3 cells and A549 cells transfected with an adenoviral CFTR expressing vector [53]. Khan et al. and Forterre et al. used HEK 293T cells to load mRNA/protein into EVs that achieved successful cellular delivery and targeting [54,55]. Unique molecular mechanisms have also been reported to induce specific mRNA/protein loading into EVs in HEK 293T models. Nedd4 family-interacting protein 1 (Ndfip1) induced EV loading of phosphatase and tensin homolog deleted on chromosome 10 (PTEN) by Nedd4 family proteins [56,57]. Using this EVs releasing mechanism, Sterzenbach et al. loaded protein into EVs. They linked Cre recombinase with WW domains of the Nedd4 binding site of Ndfip1 allowing connected WW domains to be loaded into EVs and secreted into the extracellular area [58].

#### 2.1.7. DNA

DNA modifications are mainstays of gene therapy, and recent research has significantly improved vector delivery and safety in gene therapy [59]. EVs are attractive delivery mechanisms for DNA modifications due to the higher efficiency and safety of EVs. Kanada et al. transfected Cre-encoding plasmid DNA into HEK 293T, and the successfully isolated EVs contained the plasmid DNA [17]. The same group also loaded minicircle DNA that encodes prodrug-converting enzymes into 4T1 cells and then isolated EVs containing the DNA [60]. Similarly, Haney et al. isolated EVs containing DNA from IC21 macrophages after transfecting TPP1-encoding plasmid DNA [61]. Although only a few attempts have been reported, Tran et al. demonstrated effective DNA loading conditions in transfected *E. coli* using several plasmids. They showed that a higher copying ability of the plasmid induced more DNA loading, and the plasmid origin affected loading efficiency, while plasmid size was not a significant factor in the range of 3.5 kb to 15 kb. [62]. The CRISPR/Cas9 system allows for precise DNA manipulation. This system has been commonly encapsulated through viral vectors [63]. The loading of CRISPR/Cas9 into EVs presents a novel area for improving safety and stability issues inherent to viral vectors. Li et al. successfully loaded C/EBPα CRISPR/Cas9 into EVs. [64] Since RNA-binding proteins can increase the incorporation of RNA into EVs [65], they fused RNA-binding protein HuR with exosomal membrane protein CD9 to increase the loading efficiency. To engineer the CRISPR/Cas9 cargo, they transfected CD9-HuR and CRISPR/Cas9 with packing plasmid psPAX2 and pMD2G into HEK 293T cells and isolated EVs [64].

Taken together, researchers have developed numerous cell-based loading approaches so far. For small molecules drugs, passive loading methods can be applied with anticipated efficiency. In contrast, biomolecules have to be delivered into the donor cells with active loading methods, such as transfection and vial transduction.

### 2.2. Non-Cell-Based EVs Loading Methods

**Figure 2.**
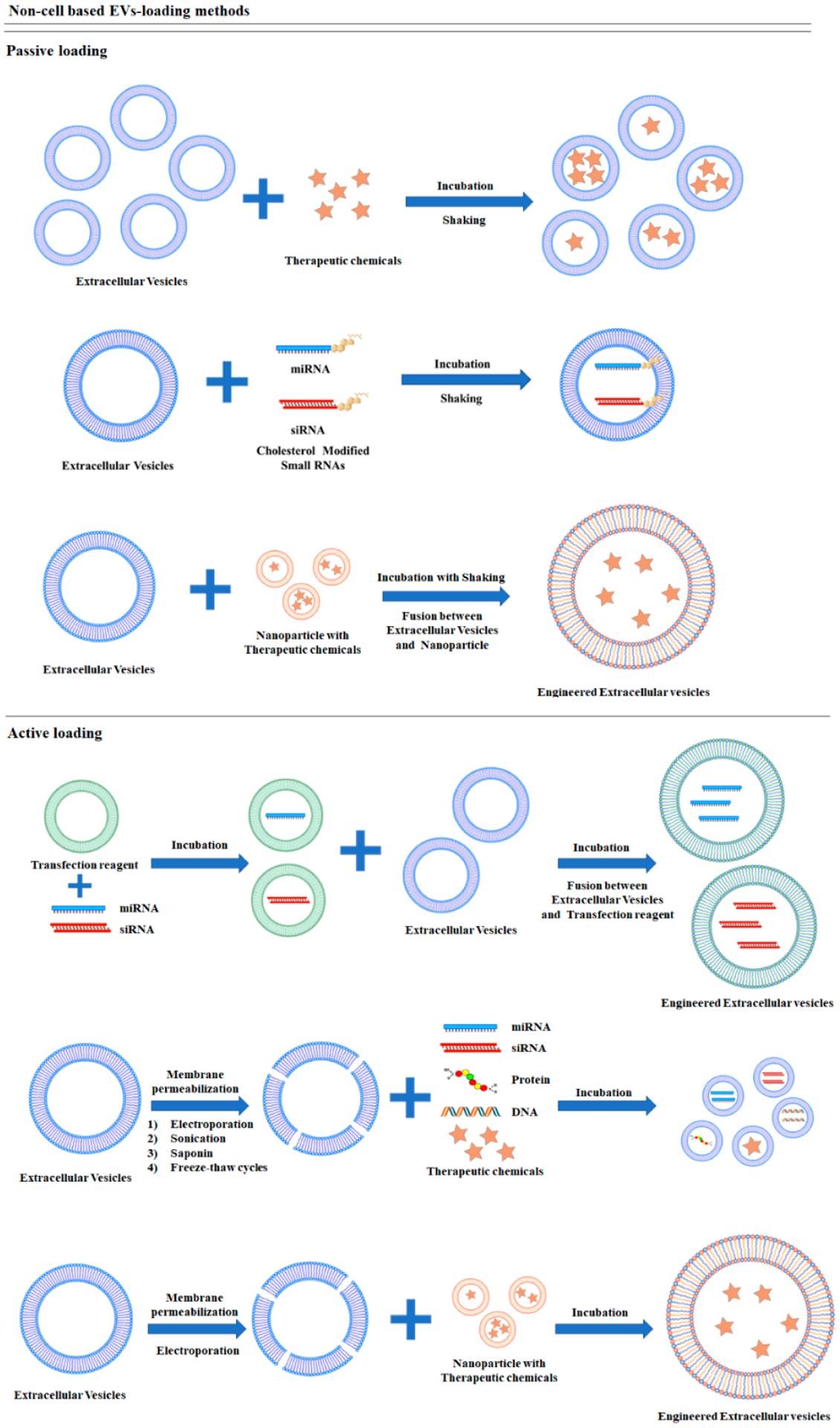
Schematic diagrams of non-cell-based cargo loading methods.

Non-cell-based EV loading methods, also known as exogenous or direct loading, deposits a therapeutic cargo into EVs after isolation. Different siRNA, miRNA, proteins, CRISPR/Cas9, hydrophobic compound derived natural product, and anticancer drugs can be loaded into EVs through sonication, electroporation, transfection reagents, and a specific buffer agent (Figure 2). Some compounds can be loaded into EVs by mixing at room temperature. Although these loading methods can be classified as passive and active loading, they also can be combined for optimized loading efficiency [66]. Passive loading involves loading the therapeutic cargo into EVs through diffusion, whereas active loading consists of the disruption of EV membranes through electroporation or sonication, allowing entry into the EVs. After completing the reaction, the membrane is recovered by incubation for a while at room temperature or 37 °C. Through this process, EVs can be stabilized [67].

#### 2.2.1. Doxorubicin

Ingato et al. and Wu et al. loaded doxorubicin into EVs derived from cancer cells by incubating 25 μg/mL EVs in 1 mL PBS (by protein content) and 1 mg/mL doxorubicin mixture at 37 °C for several hours [68,69]. Triethylamine solution has also been used for incubating EVs and doxorubicin [70,71]. Saponin induces permeabilization of the EV membrane, creating pores by removing membrane cholesterol, and can be used for loading EV cargo without destroying the membrane [72]. Goh et al. used saponin to load doxorubicin into EVs isolated from U937 cells. They compared the efficiency of several methods, including simple mixture incubation at room temperature for 24 h, simple mixture incubation at 37 °C for 24 h, saponin-added incubation at room temperature for 5 min, and three freeze-thaw cycles. Even though these methods incorporated doxorubicin into EVs, they differed in loading efficiency. When loading 200 μg/mL doxorubicin, the saponin method showed the most efficient loading (~50%) [73]. Doxorubicin was loaded into EVs derived from HEK 293T cells and HeLa cells via electroporation [74–76]. Qi et al. and Zhang et al. conjugated magnetic iron oxide with EVs to improve targeting efficiency and then mixed doxorubicin via moderating stirring incubation or electroporation [77,78]. Alternatively, Li et al. loaded doxorubicin into EVs first and then conjugated magnetic iron oxide [79]. Both methods improved loading efficiency. Srivastava et al. applied gold nanoparticles (GNPs), which combine well with small molecules but have limitations as a drug carrier, so their incorporation as an EV cargo can address the limitation. First, they conjugated GNPs with doxorubicin using hydrazone pH-sensitive linkers. After then, doxorubicin was simply incubated at 37 °C at 250 revolutions per minute (rpm) for 2 h [80]. Kim et al. and Rayamajhi et al. synthesized hybrid EVs using simple thin-film hydration and then loaded doxorubicin via sonicator [81,82].

#### 2.2.2. Curcumin

Carobolante et al. encapsulated curcumin into EVs isolated from cow skim milk and Caco-2 cells by mixing curcumin ethanol solution with EVs in PBS at room temperature overnight with stirring [83]. Tian et al. also loaded curcumin into EVs via mixing. To improve targeting, they conjugated cyclo (Arg-Gly-Asp-D-Tyr-Lys) peptide, which has a high affinity for integrin αvβ3 [84]. Zou et al. used saponin for membrane permeabilization and engineered the surface of EVs through donor cell transfection. They transfected 10E8-pDisplay expressing vector, which targets Chinese hamster ovary cells. Using EVs isolated from these engineered cells, they encapsulated curcumin into EVs via saponin permeabilization [85]. Others have used repeated freeze-thaw cycling to load curcumin [84,86]. Magnetic iron oxide nanoparticles have also been used with curcumin. Mixing EVs, curcumin, and magnetic iron oxide nanoparticles followed by electroporation successfully encapsulates them into EVs [87].

#### 2.2.3. Paclitaxel

Paclitaxel is an anticancer drug that inhibits cell division, but poor cellular uptake and side effects limit its efficacy. Several EV loading methods of paclitaxel can improve therapeutic efficiency and minimize side effects. Saari et al. and Agrawal et al. loaded paclitaxel into EVs via simple incubation [88,89]. Sonication successfully loads paclitaxel, as demonstrated by Kim et al. and Salarpour et al. in which paclitaxel and EVs were mixed and sonicated [90,91]. In EVs derived from IC21 macrophages, sonication resulted in roughly 30% more loading efficiency than saponin permeabilization [61]. A similar tendency was confirmed by others in EVs from RAW264.7 cells when evaluating with mixing and EV incubation, electroporation, and sonication. For the incubation, the mixture was incubated at 37 °C with 1 h of shaking. The mixture was electroporated at 1000 kV for 5 ms and incubated at 37 °C for 30 min to promote recovery of the exosome membrane. The mixture was sonicated for 6 cycles of 30 s on and off with 2 min of cooling between each cycle and then incubated at 37 °C for 60 min for exosome membrane recovery. The loading efficiency of paclitaxel into EVs was as follows: simple incubation < electroporation << sonication [81].

#### 2.2.4. Aspirin

Delivering functional aspirin to target cells is difficult due to poor water solubility [92]. These limitations have prevented aspirin use in severe diseases such as cancer, even though its effects as an anticancer agent have been long known. To resolve the low water-solubility issue, Tran et al. loaded aspirin into EVs for cancer treatment. Before loading aspirin into EVs, they constructed a nanoparticle of poloxamer 407 with MgO or TPGS. This nanoparticle was mixed with aspirin and EVs isolated from HT29 and MDA-MB-231 cells and incubated at room temperature for two hours with shaking [93]. Aspirin has also been loaded into EVs via freeze-thaw cycling with incubation and sonication [92]. Kalinec et al. loaded aspirin into EVs isolated from auditory HEI-OC1 cells. They incubated EVs and aspirin at 25 °C for 1 h with 5 min sonication [94].

#### 2.2.5. Other Drugs

Yang et al. reported a method for the loading of the antibacterial linezolid into EVs derived from RAW264.7 cells. Linezolid was mixed with EVs, and the mixture was incubated at 37 °C for 1 h [95]. Dexamethasone was successfully loaded into EVs isolated from HEI-OC1 cells by room temperature incubation and 5 min of sonication [96]. Using this protocol, they loaded various drugs, including aspirin, arachidonic, eicosapentaenoic, docosahexaenoic, and linoleic acids, lipoxin A4, and resolvin D1 [94]. Erastin is a chemotherapeutics drug inducing ferroptosis. Even though erastin is a small molecule drug, poor water solubility and nephrotoxicity limit its applications. Similarly, Yu et al. encapsulated erastin into EVs to treat triple-negative breast cancer using sonication [97].

#### 2.2.6. miRNA

EVs can be loaded with miRNA for safe and efficient delivery to target cells. For incorporating miRNAs into EVs, liposome-based transfection reagents can be applied to isolated EVs [98]. miRNAs, miR-335-5p, miR-21, and miR-143 have been successfully transfected into EVs with transfection reagents [99–101]. Electroporation is one of the most commonly used methods for loading miRNA directly into EVs. For example, miR-155, miR-26a, miR-155, miR-31, miR-451a, miR-939, miR-128-3p, miR-223 mimic, and miR-142 mimic have been successfully loaded [102–108]. Several methods have been devised to improve loading efficiency. Zhang et al. developed a method to load miRNA into EVs using modified calcium chloride transfection. EVs, miRNA mimic, and calcium chloride were mixed with PBS and placed on ice for 30 min, followed by miRNA incorporation by heat shock at 42 °C for 60 s [109]. According to the miRNA copy number per EV, the loading efficiency of modified calcium chloride transfection is comparable with the electroporation [110]. It has been reported that a cholesterol group is attached to the 3′ end of the miRNA to facilitate in vivo delivery [111]. Interestingly, Gong et al. identified that cholesterol-modified miRNA can quickly internalize into EVs by itself due to the hydrophobic-moiety modification. They incubated cholesterol-modified miRNA with EVs in PBS (37 °C, 90 min, 500 rpm shaking). The loading efficiency of miR-159 was 1.2%, whereas cholesterol-modified miR-159 improved to 5.33% [70]. Zhang et al. used peptide-conjugated EVs as well as cholesterol-modified miRNA. To improve the targeting property of EVs, they conjugated cyclo (Arg-Gly-Asp-D-Tyr-Lys) peptide [c(RGDyK)], which can detect ischemic brain, onto the surface of EVs. Then, they incubated the engineered EVs with cholesterol modified miR-210 in PBS (37 °C, 60 min) and successfully obtained the miR-210-loaded EVs for therapy [112]. Jeyaram et al. loaded miRNA by pH gradient EV modification. EVs were isolated from HEK293T cells and dehydrated, followed by incubation in citrate buffer of various pH for one hour. After, EVs were dialyzed in HEPES buffered saline (pH 7.0) for 24 h. Modified EVs were incubated with miR-93 in PBS at various time points and temperatures. In a citrate buffer with a pH of 2.5, a two-hour incubation with 22 °C PBS resulted in the most efficient loading of miR-93 [12].

#### 2.2.7. siRNA

Similar as miRNA, non-cell-based EV loading methods are widely used for siRNA loading by researchers. Some studies reported that transfection reagents promote siRNA loading into EVs. After isolating EVs, siRNA was transfected with EVs using commercial transfection reagents. EVs, siRNA, and reagents were incubated together for several hours at 37 °C allowing siRNA incorporation in EVs. [113,114]. Another popular method of loading siRNA directly into EVs is electroporation. The external electric field increases cellular membrane permeability by creating a hydrophilic pore and allows siRNA to enter EVs. This method has allowed EVs to knock down gene expression in target cells using siRNA cargo, including several genes, GAPDH, cyclophilin B, BACE1a, BACE1b, luciferase, PLK-1, VEGF, Kras^G12D^, and TPD52 [115–120]. Some studies have attempted to increase the silencing efficiency by allowing siRNA to reach target cells with high probability. They conjugated EVs with peptides that bind specific receptors of target cells. The transfection of donor cells can create EVs with specific peptides within their membrane. Rabies virus glycoprotein (RVG)-derived peptide, which binds p75 neurotrophin receptor, was conjugated to EVs, and then siRNA was loaded through electroporation [1,121,122]. Chen et al. conjugated klotho protein to EVs, which binds circulating endothelial progenitor cells (EPCs). Then, adenosine kinase (ADK) siRNA was loaded into EVs through electroporation to develop a cardiovascular disease therapeutic [123]. However, studies found electroporation can induce siRNA aggregation, which may cause overestimation of loading efficiency [124,125]. Lamichhane et al. loaded siRNA into EVs using a water bath sonicator by mixing EVs and siRNA in PBS, incubating for 30 min at room temperature, followed by sonication at 35 kHz for 30 s. Sonication of more than 30 s degraded the EVs [126]. Liu et al. loaded opioid receptor Mu (MOR) siRNA into RVG-EVs by sonication, and sonication achieved higher efficiency than electroporation [127]. Milk-derived EVs can inhibit cell growth of various cell lines [128], and several studies have used milk EVs loaded with siRNA for anticancer effects. After isolating milk-derived EVs, siRNA can be loaded with transfection reagents [129–132]. Cholesterol-conjugated hydrophobic siRNA can be loaded into EVs by simple incubation [11,133]. Biscans et al. conjugated several natural lipids, including fatty acids, sterols, and vitamin E, with siRNA. They suggested that vitamin E-conjugated siRNA showed the most efficient loading and delivery to neurons [134]. Haraszti et al. elucidated that the type of linker used to conjugate cholesterol to hydrophobic siRNA affected loading efficiency. For the huntingtin siRNA targeting neurons, the TEG (triethyl glycerol) linker showed better efficiency than the C7 (2-aminobutyl-1-3-propanediol) linker [135]. Jhan et al. fused several lipids such as DOTAP, POPC, DPPC, and POPG with EVs to make a hybrid-lipid membrane structure and then loaded siRNA into EVs through electroporation. These engineered EVs showed a higher gene silencing effect than lipofectamine transfection [136]. Jeyaram et al. loaded GAPDH siRNA through pH gradient modification of EVs. HEK 293T-derived EVs were incubated in pH 2.5 citrate buffer for one hour. Modified EVs were incubated with siRNA in PBS for 2 h at 22 °C. The modified EVs showed 54% knockdown efficiency, whereas unmodified EVs did not decrease GAPDH expression level [12].

#### 2.2.8. Protein

Haney et al. loaded recombinant human TPP1 protein into EVs derived from IC21 macrophages through sonication or saponin permeabilization methods. For the sonication method, 500 μL EVs suspension (10^11^ particles per mL) were supplemented with 5 μL TPP1 (20 μg/100 μL) and sonicated at room temperature for 30 min. For the saponin permeabilization, 500 μL EVs suspension (10^11^ particles per mL) were supplemented with 5 μL TPP1 (20 μg/100μL) and 10 μL saponin solution to final concentration 0.4 mg/mL, and incubated at RT for 30 min. Although sonication loaded 40% more protein than saponin permeabilization, both methods loaded 7-times and 5-times more than donor cell transfection, respectively [61].

#### 2.2.9. DNA

Exogenous DNA can be loaded into EVs via electroporation. Lamichhane et al. isolated EVs from HEK 293T cells and loaded 5 μg of dsDNA into 10 μg of EVs in a final volume of 50 μL electroporation buffer [19]. Kao et al. isolated EVs from CHRF cells and loaded Cy5-labeled pGFPns via electroporation [137]. Jeyaram et al. used HEK 293T cell EVs after incubating in pH 2.5 citrate buffer for one hour. Modified EVs were mixed with single-stranded DNA in PBS for 2 h at 22 °C [12]. Kim et al. isolated EVs from HEK293 and SKOV3 cells and loaded CRISPR/Cas9 targeting PARP-1 through electroporation. A total of 10 μg of DNA was mixed with 30 μg of exosomes and Cas9- and sgRNA-expressing plasmids were loaded by electroporation (1000 V, 10 ms, 2 pulses) [138].

Comparing with the cell-based methods, non-cell-based loading approaches have more flexibility without affecting the donor cells. Nevertheless, these methods may alter the integrity of the EV membrane and affect the bioactivity of cargos depending on the approaches (Table 1).

## 3. Conclusions and Future Directions

Dysregulated gene expression promotes the pathogenesis of many human diseases. Gene therapy is considered an ideal treatment for various disorders, such as inherited diseases and many cancers [139]. However, the immune rejection response against foreign substances and the limited stability of the vectors limits optimal application [140]. Artificially engineered nanoparticles, such as liposomes, polymeric nanoparticles, and magnetic nanoparticles, have been widely used for drug delivery. Nanoparticles have many applicable properties, such as high loading efficacy, safety, and cell targeting benefits as target-specific drug carriers. Even though nanodrugs are being investigated in clinical trials, the immune responses, toxicity issues, and high cost have limited development [141]. However, the EV delivery system has distinct advantages over vector delivery and artificial nanoparticles. EVs are natural products originating from the cell surface, and thus better at avoiding activating immune responses [142]. Another advantage of EVs is specificity for target cells. The tissue-homing characteristics of EVs allow the transport of their therapeutic cargo to distant target cells [143]. In diseases where drug receptors lack sufficient expression due to mutations or situations involving biological barriers such as the blood-brain barrier, the drug-loaded EVs can facilitate drug delivery by fusing with the membrane of target cells [144]. For example, many have studied the treatment of triple-negative breast cancer, which lacks estrogen receptors, progesterone receptors, and human epidermal growth factor receptor 2 [67,145]. EVs present a targeting system that could overcome this kind of barrier for treatment. For this reason, they are expected to be safer and more efficient for delivering oligonucleotides and plasmids [146].

Despite these advantages, there are several developmental challenges. Although several methods for isolating EVs, such as ultracentrifugation, immunoaffinity, size exclusion, precipitation, and microfluidics techniques, have been reported, there are still no efficient isolation methods to achieve pure EVs. Jeppesen et al. reassessed the characteristics of EVs reported by other research groups. They showed at least 20 different results from existing reports [4]. Another disadvantage is the diverse mechanisms of cellular uptake, depending on the cell type and administration method [147]. Some researchers conjugated EVs with functional ligands to target the cells to improve specific cellular uptake [1,112,121,122]. Sometimes loading methods affect EV stability. The integrity of EVs can become damaged by sonication or permeabilization agents [66]. Additionally, electroporation has been known to aggregate siRNA during the loading process. To improve this technical problem, Kooijmans et al. added EDTA to the electroporation buffer [125]. Even though the effect of a low amount of EVs cargo is similar to that of conventional methods, the loading efficiency may be improved for better results [148]. To increase the loading efficiency, several researchers conjugated nanovesicles, which have high loading capacity [23,77,78,87,149]. Many challenges remain in designing the most efficient and effective EV loading methods.

The field of EVs is exponentially growing, as many studies focus on technical optimization of loading methods in the pre-clinical stage. In this review, we mainly summarized the methods for cargo loading into naturally generated EVs. However, some engineered EVs, such as gesicles [150], artificial EVs [151], and mimetic EVs [152] are not discussed in the current review.

Human cell/tissue-derived EVs have been applied in clinical trials as anti-cancer drug vehicles [153,154]. For instance, in a clinical trial (NCT03608631), KrasG12D siRNA was proposed to be loaded into mesenchymal stromal cells (MSC)-derived EVs for the treatment of patients with metastatic pancreas cancer. Besides the cancer studies, in the clinical trial NCT03608631, miR-124-enriched EVs from MSC cells were proposed to treat acute ischemic stroke.

Although safety and tolerance of EVs as carriers have been observed in most clinical trials [153–156], many challenges remain before applying EVs as drug carriers in clinical trials, such as loading efficiency and the purity of EVs for clinical use. Little is known about the mechanisms of cell-based or non-cell-based drug loading methods. Therefore, further investigations are expected not only to develop novel methods but also to explore the molecules that improve cargo loading efficiency through mechanistic studies.

## Figures and Tables

**Table 1.** Comparison of cargo loading methods.

Cell-Based Cargo Loading Methods				
Method	Cargo	Pros	Cons	Reference
Passive incubation	Small molecule drugs, such as doxorubicin and curcumin	Simple and convenient; can be adopted by most laboratories	Low loading efficiency; may affect the EV components	[22–25]
Transfection	Biomolecules, including small RNAs, mRNA, DNAs and proteins	Load large cargos; efficiency is relatively high	Transfection reagent is required; loading efficiency relies on the transfection efficiency	[34–37,52,53,64]
Non-Cell-Based Cargo Loading Methods				
Method	Cargo	Pros	Cons	Reference
Passive incubation	Small molecules drugs, such as doxorubicin and curcumin	Very simple; can be easily adopted by most laboratories	Low loading efficiency	[68–71,83,93]
Transfection	MiRNAs and siRNAs	Load large cargos; enhanced efficiency	May alter the property and structure of EVs	[98,113,114]
Electroporation/Sonication/Freeze-thaw/Saponin	Small molecules drugs; biomolecules, including small RNAs, mRNA, DNAs and proteins	Possible to load macromolecules; loading efficiency is relatively high	Increase EV instability; small RNA or EV aggregation; additional equipment or wash step may be required	[61,84,86,87,90,91, 102–108,137,138]
